# The gap in capacity building on climate, health, and equity in built environment postsecondary education: a mixed-methods study

**DOI:** 10.3389/fpubh.2023.1090725

**Published:** 2023-07-27

**Authors:** Adele Houghton

**Affiliations:** ^1^Biositu, LLC, Houston, TX, United States; ^2^Harvard TH Chan School of Public Health, Boston, MA, United States

**Keywords:** climate change, population health, social equity, built environment, transdisciplinary curriculum

## Abstract

Institutions of higher education are feeling increasing pressure from both students and the international climate community to offer more courses and joint degrees on the role of the built environment in advancing climate action, population health, and social equity. The built environment plays a leading role in this new, transdisciplinary approach. Thoughtfully designed buildings, neighborhoods, and communities can simultaneously lower *per capita* greenhouse gas emissions, reduce population exposure to dangerous climate-sensitive extreme weather events, reduce disparities in climate-related health outcomes, and advance social equity goals. This mixed-methods study explored the extent to which post-secondary courses and joint degree programs teach students the research methods and technical skills they will need to design and implement built environment interventions addressing the effects of climate change on population health and social equity. The study found that the number of universities offering courses addressing climate, health, and equity in the built environment grew from 2018 to 2022. The number of joint planning/public health degree programs rose from four in 2005 to 15 in 2022. No joint architecture/public health degree programs were identified. A detailed review of 99 course descriptions from three universities found that 17 courses (roughly 1/5 of the total) covered population health, built environment, and climate change; and, 2/3 of the set (*n* = 60) covered two out of the three topics. Schools of public health were more likely to offer courses covering all three topics, whereas schools of architecture were more likely to include the building scale in relevant courses. Exposure pathways and social equity/vulnerable populations were the most common methods included in relevant courses. Professors and administrators at institutions identified by the study as “transdisciplinary-ready” reported that accreditation requirements and university rules governing the allocation of student tuition had slowed efforts to offer cross-listed courses. However, faculty in these institutions regularly collaborate informally with their peers – both on transdisciplinary research and by offering guest lectures in each other’s courses. The results of this study show that, while universities have made great strides over the past 18 years in beginning to support transdisciplinary research and pedagogy, institutional barriers and gaps in key content areas remain.

## 1. Introduction

Institutions of higher education are feeling increasing pressure from both students and the international climate community to offer more courses and joint degrees on the role of the built environment in advancing climate action, population health, and social equity.

Young people today experience climate change in real time. No longer is it spoken of as a phenomenon that may happen in the distant future to animals in remote locations. Each year is punctuated by deadly heat waves, wildfires, floods, and storms that result in destroyed communities and loss of life. No one is immune from experiencing climate change anymore – regardless of geographic location or income level (1, 2). Growing evidence shows that high school and college students not only consider climate change an existential threat to the future of humanity but also view it as a complex challenge spanning numerous disciplines and affecting society as well as natural ecosystems. For example, Hickman et al. found that over half of children and young people aged 16–25 who participated in a global survey about climate anxiety reported feeling “very” or “extremely” worried about climate change. Over half also responded negatively when asked whether the government in their country “was taking [young people’s] concerns seriously,” was “doing enough to avoid a climate catastrophe,” or “was acting in line with climate science” (3).

Many students bring that anxiety, commitment, and transdisciplinary lens with them when they enter university with the goal of dedicating their lives to advancing solutions to the climate crisis. Too often, what they encounter is an institution that is designed to facilitate the creation of deep levels of knowledge on narrow topics but limited infrastructure supporting research into complex systems that cross traditional disciplinary boundaries.

### 1.1. Transdisciplinary pedagogy: background and relevance to curricula addressing the intersection of climate, health, and equity in the built environment

The concept of “transdisciplinarity” is relatively new. It was first publicly debated in 1970 at a seminar in Nice, France hosted by the Organization of Economic Cooperation and Development (OECD) and the French Ministry of Education (4, 5). That same year, a PhD student in human behavior sciences, Jack Lee Mahan, Jr., published a dissertation on the topic (5, 6). In both cases, the term was proposed as an alternative to the status quo amid a backdrop of university students protesting traditional, discipline-specific pedagogy and the publication of environmental treatises like *The Limits to Growth* (1972) (7), which questioned whether using linear concepts like “progress” to motivate discipline-specific scientific questions would eventually lead to ecosystem collapse (5).

The term fell out of usage during the economic and social retrenchment in Western countries that followed the economic crisis in 1973 caused by the Organization of Arab Petroleum Exporting Countries (OAPEC) oil embargo (5). Its return to prominence in the 1990’s coincided with the rise of three complex, global challenges: (1) the end of the Cold War (which prompted a reorganization of the world order), (2) increasing concern about the effects of globalization on national economies and labor conditions, and (3) new urgency around environmental sustainability and climate change sparked by the 1992 United Nations Earth Summit in Rio de Janeiro (5).

Tress et al. (8) and Morton et al. (9) distinguish transdisciplinary research from other types of research as follows. Disciplinary research remains within a single academic tradition in all respects, including the development of the research question, use of theoretical frameworks and methods, and communication of results. Multidisciplinary research involves more than one academic discipline in developing the research goal, but each discipline conducts its assigned piece independent of the others. Participatory research involves knowledge exchange between academic and non-academic stakeholders, but all research activities remain separated (either taking place on the academic side or on the non-academic side). Interdisciplinary research crosses disciplinary boundaries to create shared knowledge, but the process remains entirely within the academy. Transdisciplinary research brings real world questions into academic settings by involving both academic researchers and practitioners and/or community members. In this way, transdisciplinary research is best positioned both to address complex, global challenges like climate change and to center social equity in the formulation of the research question and (ultimately) the recommended intervention.

As key producers of knowledge, universities have naturally found themselves at the center of the debate around transdisciplinary research and pedagogy – starting with student frustration in the late 1960’s and early 1970’s around the way universities were organized and taught academic subjects (10). More recently, Scholz (10) proposed transdisciplinarity as an alternative to the “Triple Helix” and “Third Mission” approaches to university research, both of which he rejects as overly profit-driven. Triple Helix involves an alliance between university, industry, and government. Third Mission refers to entrepreneurial partnerships between universities and private practice. Scholz proposes transdisciplinarity as a pathway that would center advancement of the public good in all research by authentically collaborating with practitioners and other stakeholders who have real-world experience working on complex projects spanning multiple disciplines. Scholz calls this approach “science with society,” which he distinguishes from the current system which he calls “science for society” (10).

Daneshpour and Kwegyir-Afful (11) found in a scoping review of transdisciplinary curricula drawn from over ten countries that sustainability courses dominated the list. They hypothesized that the multidisciplinary nature of the United Nations Sustainable Development Goals (12) and the emphasis that is often placed on real world scenarios in sustainability courses are two reasons sustainability curricula are disproportionately likely to follow transdisciplinary pedagogy. Seidl et al. (13) go so far as to claim that transdisciplinarity has become the “consensus” pedagogy among sustainability science programs. Examples of transdisciplinary sustainability courses and trainings include the AGE-WELL (Aging Gracefully across Environments using Technology to Support Wellness, Engagement and Long Life NCE Inc.) training in Canada (14), the “Sustainability Challenge” course in Vienna, Austria (15), and a transdisciplinary master of engineering program at Texas Tech University in the US (16). Velez, Hall, and Lewis’s public policy course at Virginia Tech in the US further illustrates the strong affinity between sustainable design research and practice and transdisciplinarity – beginning with the course’s name, “SuperStudio.” The course follows a similar process to architecture design studio courses, which use project-based learning to iterate between research and design to solve a specific challenge and often include feedback from community stakeholders who will be impacted by the project (17).

This study explores the extent to which transdisciplinary pedagogy in architecture, planning, and public health schools addresses the intersection of climate change, population health, and social equity in the built environment, because that nexus represents a powerful leverage point in the climate crisis that could be activated using a transdisciplinary approach.

### 1.2. Definition of terms

**Transdisciplinary pedagogy**: the exact definition of transdisciplinarity continues to evolve (5, 8, 9, 18). However, several common elements reflect its roots in social justice and focus on complex, global challenges. For the purposes of this study, transdisciplinary pedagogy is defined as an approach to teaching that integrates multiple disciplines into a single course or degree program and includes practice-based learning.

The following definitions are provided to set a common understanding for the remainder of the paper.

**Architecture** (19): the art and practice of designing structures, including their relationship with the surrounding built and natural environment. The practice of architecture includes coordinating allied disciplines (such as engineers, landscape architects, and contractors) to develop a set of project documents that will protect the health, safety, and welfare of future building occupants and society.

**Built environment** (20): “a general term covering residential, industrial, and public buildings, roads, and services, such as water supplies, electrical wiring, and sewerage in human settlements.”

**Climate change** (21): “a change of climate which is attributed directly or indirectly to human activity that alters the composition of the global atmosphere and which is in addition to natural climate variability observed over comparable time periods.”

**Climate change mitigation** (22): “a human intervention to reduce emissions or enhance the sinks of greenhouse gases.”

**Climate change adaptation** (22): “the process of adjustment to actual or expected climate and its effects, in order to moderate harm or exploit beneficial opportunities.”

**Climate change resilience** (22): “the capacity of interconnected social, economic, and ecological systems to cope with a hazardous event, trend or disturbance, responding or reorganizing in ways that maintain their essential function, identity and structure.”

**Population health** (23): “the health outcomes of a group of individuals, including the distribution of such outcomes within the group.”

**Public health** (20): “an organized activity of society to promote, protect, improve, and when necessary, restore the health of individuals, specified groups, or the entire population.”

**Social equity** (24): “social equity implies fair access to livelihood, education, and resources; full participation in the political and cultural life of the community; and self-determination in meeting fundamental needs.”

**Urban planning** (25): the process of planning, designing, and developing the physical, social, and economic aspects of urban space, primarily concerned with improving the quality of life for residents.

### 1.3. Built environment’s central role in population health equity and the health effects of climate change

The built environment plays a leading role in setting the context for population health and the ways in which different segments of society experience climate change. Buildings are responsible for almost 40% of global greenhouse gas emissions. Adding the allied fields of transportation and land use raises the estimate to 60% worldwide (26). Buildings are also overwhelmingly the location where populations shelter during climate-sensitive extreme weather events. Also, many of the underlying social determinants of health (27, 28) that increase the risk of poor health outcomes after exposure to such events (29) are influenced by building design and land use (30). For example, an individual who lives in a neighborhood lacking sidewalks, parks, and a healthy grocery store is at higher risk of obesity, diabetes, and hypertension (31, 32). Lack of green space is also a risk factor for exposure to extreme heat and flooding (33, 34). Individuals diagnosed with obesity, diabetes, and hypertension are at higher risk of negative health outcomes when exposed to those two climate-sensitive extreme weather events (29, 35). If that individual’s home is located in a flood plain or near a source of air pollution (such as a freeway), not well insulated, under-airconditioned/ventilated, and/or fitted out with building materials that off gas toxins, spending time in the home during a heat or flooding event could exacerbate symptoms arising from exposure to the event itself. Thoughtfully designed buildings, neighborhoods, and communities – on the other hand – can simultaneously lower *per capita* greenhouse gas emissions, reduce population exposure to dangerous climate-sensitive extreme weather events, and advance social equity goals such as reducing disparities in climate-related health outcomes (36, 37).

In spite of the built environment’s central role in both the cause of climate change and its effects on human health, that link has been largely excluded from postsecondary education. This is particularly true at the building scale. Architecture, landscape architecture, and building engineering accreditation boards and professional core competencies continue to treat climate change mitigation as an abstract estimate of carbon equivalent emissions and resilience as a question of protecting infrastructure and property value (38–42). In both cases, the health needs of the population that the buildings were designed to serve are excluded from the conversation. Similarly, when courses tailored to the fields of public health and medicine teach students about the role of the built environment in climate, health, and equity, they either focus on healthcare facilities or zoom out to large scale community planning questions like greenways and urban sprawl, glossing over the crucial question of how a community plan translates into building design (43–45). While both of these approaches touch on important aspects of the topic, they do not prepare future leaders in real estate, design, and public health to effectively address the effects of climate change on population health and social equity – particularly at the local level where the majority of these activities take place. This paper argues that a transdisciplinary pedagogical approach is required to achieve that goal. It is necessary to teach students how to integrate tools and frameworks from multiple disciplines to address the complex adaptive challenges they will face in their post-graduate careers – whether in research or practice.

**Figure 1:**
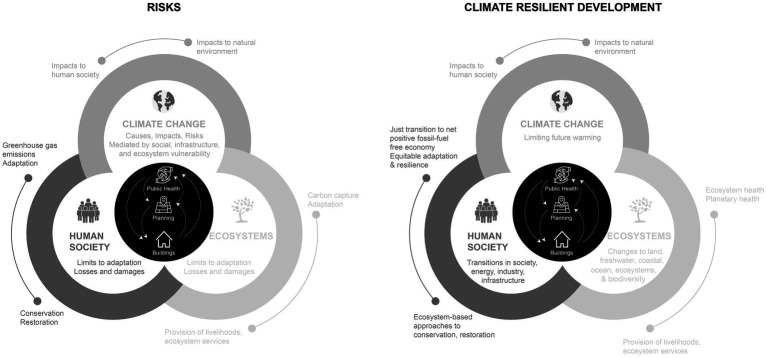
Interrelations between buildings, planning, and public health related to climate risks and resilient development. Adapted from Figure SPM.1, IPCC, 2022: summary for Policymakers (46).

The Intergovernmental Panel on Climate Change’s (IPCC) Sixth Assessment reports (1, 2) reflect the shift in the scientific consensus on how to study and act on climate change. Instead of dedicating the majority of the report to summary descriptions of individual phenomena, the report’s authors highlight examples of how the scientific community and their partners in government and the private sector are moving towards transdisciplinary research and implementation science – with a particular emphasis on how human and natural systems intersect and influence each other. Figure 1, adapted from the IPCC report, illustrates how public health, architecture/real estate development, and planning interact with each other both in contributing to the current climate crisis and in working together to stop emissions and protect population and planetary health.

This mixed-methods study explores the extent to which academic institutions are responding to pressure from both students and the international climate community to build capacity in this area. It asks the following research question: how widespread are postsecondary courses and joint degree programs that teach students the research methods and technical skills they will need to design and implement built environment interventions addressing the effects of climate change on population health and social equity?

## 2. Materials and methods

### 2.1. Background: synthesizing existing model curricula and core competencies into key elements for transdisciplinary curricula addressing climate, health, and equity in the built environment

This study was motivated by a preliminary review of the literature conducted in fall 2022 on model curricula and key competencies at the intersection of climate change, public health, and the built environment (e.g., architecture and planning) – particularly in relation to advancing social equity. A query in Google Scholar for “curriculum + climate change + health + built environment” returned two relevant entries, the seminal 2009 paper “A Model Curriculum for a Course on the Built Environment and Public Health: training for an Interdisciplinary Workforce” by Botchwey et al. (47) and a qualitative study from 2020 assessing the pedagogical strengths and lessons learned from a pilot course based on Botchwey et al.’s model curriculum that was targeted to architecture and landscape architecture students at an historically black university (HBCU) in the US (48).

Botchwey et al. (47) builds off of the 2005 report *Promoting Interdisciplinary Curricula and Training in Transportation, Land Use, Physical Activity, and Health* by Sclar et al. (49). Both papers scanned existing curricula at US postsecondary institutions and contacted instructors for additional information about active courses. Sclar et al. developed an “ideal” curriculum derived from a scan of coursework in all 70 accredited urban and community planning schools on the Association of Collegiate Schools of Planning (ACSP) list at the time of the study. Botchwey et al. synthesized overarching themes and best practices from six courses in US postsecondary institutions that were offered in either planning schools, public health schools, or cross-listed in both. Table 1 summarizes both papers’ recommendations.

**Table 1:** Key elements for transdisciplinary curricula addressing climate, health, and equity in the built environment.

Promoting interdisciplinary curricula and training in transportation, land use, physical activity, and health (2005) (49)	A model curriculum for a course on the built environment and public health: training for an interdisciplinary workforce (2009) (47)	Global Consortium on Climate and Health Education (GCCHE) Climate & Health Core Competencies for Health Professions Students (2020) (50)
**History**: Historical perspectives on urban planning and public health **Theory**: Concepts and theories in transportation, land use, and population health **Methods**: Research designs, methods, and approaches for studying the effects of the built environment on population health **Tools**: Research and policy tools used to intervene on the built environment to improve public health	**Planning and public health foundations:** Planning history, Public health history, Interdisciplinary applications **Natural and built environments:** Land use and transportation, Planning design approaches, Health impact assessments, Environmental-impact assessments, Indoor and outdoor air quality, Water quality, Food security **Vulnerable populations and health disparities:** Groups who are at higher risk of disparities in health outcomes, Mental health, Social capital, Environmental justice **Health policy and global impacts:** Health policy, Sustainable planning and global warming, Healthy housing **Integration:** Final portfolio	**Domain: Knowledge and Analytical Skills** Define climate drivers (both natural and human-caused), weather, climate change, and climate variability. Identify the health impacts of climate change and effective responses on the part of specific health services. Apply knowledge of levels of prevention, climate mitigation and adaptation, and explain health co-benefits of actions. Describe public health and its determinants. Apply knowledge of emergency planning skills. Access and interpret relevant local, regional, national, and global information about climate change effects on health. Apply knowledge of the ethical, professional, and legal obligations relevant to climate and health. Demonstrate understanding of the scientific consensus on climate change and concept of evolving science. **Domain: Communication and Collaboration** Demonstrate effective communication with stakeholders about climate and health topics. Work collaboratively and across disciplines on climate and health issues. **Domain: Policy** Explain the role of subnational, national and global policy frameworks and governance structures to address health risks associated with climate change. Explain climate-health activism and policy engagement roles of health professionals. **Domain: Public Health Practice Competencies** Apply climate and health knowledge to improve decisions about public health services, and adapt and improve population health. Apply knowledge of the connection between habitat and biodiversity loss and infectious diseases. **Domain: Clinical Practice Competencies** Describe ways that health care professionals and facilities can prepare for and respond to climate related health risks. Apply knowledge of climate and health to clinical care of patients.

*The text in this table is a direct quotation from each of the cited documents.

While climate change is mentioned in passing in both model curricula, it is not integral to either. The third column in Table 1 fills that gap by summarizing the Global Consortium on Climate and Health Education (GCCHE) core competencies for postsecondary courses addressing the links between climate change and health at health profession schools (50).

I synthesized the concepts in Table 1 and added two missing topics: (1) a review of key elements in courses delivered by schools of architecture and (2) the links between climate change, the built environment, and population health (particularly health disparities). The result is a list of five key elements that appear to be fundamental to developing a successful transdisciplinary curriculum addressing climate, health, and equity in the built environment:

**Exposure pathways**: it is important to map the way environmental exposures reach their target within a population or within an individual’s body, so that interventions (such as building design and land use configuration) can be tailored to interrupt negative exposure pathways and promote behavior that leads to optimal physical and mental health and wellbeing. Climate change-related exposure through the built environment often occurs across multiple pathways simultaneously. For example, when Hurricane Harvey struck Houston, Texas, US, in 2017, it set in motion exposure pathways related to flood risk (and, later, exposure to mold), extreme heat, and waterborne disease/toxins. The storm shut off power both to local residents and to 11% of US oil refining capacity, which both left residents without access to air conditioning during the heat of the summer and resulted in higher gas prices regionally and nationally. 43 US EPA Superfund sites and local wastewater treatment plants were flooded during the event, releasing toxic chemicals and pathogens into flood waters that came in contact with residents and first responders (51). Understanding the potential exposure pathways associated with the range of climate change-related hazards that might be relevant to a building site or neighborhood is the first step in designing a project that makes the best use of its location to meet net zero goals and maximize its contribution to community resilience.

**Social determinants of health (SDOH)** (28) are defined as the underlying social, economic, environmental, and political systems that contribute to disparities in health outcomes among different segments of the population. Discriminatory land use decisions like redlining (e.g., the historic practice of denying bank loans to property owners in majority non-white neighborhoods) have left a legacy of economic and health disparities in the US. Studies have shown that people living today in neighborhoods that were redlined in the 1930’s are more likely to experience lower levels of vegetation (which protects from extreme heat events) (52), lower levels of home ownership (53), and lower access to healthy food options (54), among other disadvantages that can harm their health and wellbeing. Understanding the SDOH of the population that will be served by a building or neighborhood and how their circumstances increase or decrease their risk of negative health outcomes after exposure to a climate-sensitive extreme weather event should be a fundamental component of the site assessment/scoping exercise for any building or neighborhood project, so that the design can prioritize interventions that promote health and wellbeing – particularly for those most at risk of negative health outcomes.

**Equity/vulnerable populations**: some groups in society are at higher risk of negative health outcomes either because of physiological characteristics (such as very young children and elders) or as a result of the social determinants of health (55). Buildings, land use configuration, and transportation systems can be designed to both protect vulnerable groups from exposure to climate change-related hazards and encourage health-promoting behaviors. For example, the High Point residential development in Seattle, WA, US, actively involved existing residents in the design process. As a result, the final design combines green space, walking paths, and a community garden for adults at risk of cardiovascular disease (56) with so-called “Breathe-Easy” homes targeted to the high percentage of children living with asthma in the existing public housing development (57). The combination of environmental exposures and vulnerable populations changes from one neighborhood to the next. It is therefore important to include an assessment of the current and likely future interactions between environmental exposures and population health needs on and around a building site or community plan prior to setting design goals, so that the design can respond to its context. Many times, this sort of assessment will require expertise in qualitative methods such as participatory action research (58, 59), so that community needs and priorities are centered in the final design.

**Epidemiology/biostatistical methods** (60): these methods are fundamental to applying an evidence-based and data-driven approach to building design and community/urban planning. They make it possible to estimate the relevant strength of association between a set of environmental exposures, potential health outcomes, and the role of building or land use design as a mediating factor. Quantitative methods are often used to explain the links between exposure pathways and vulnerable populations, as well as how certain design strategies could protect vulnerable populations from exposure to climate change-related hazards – such as air pollution, flooding, extreme heat, etc.

**Geospatial analysis/GIS methods** (60): spatial analysis makes it possible to estimate which environmental health exposures are more relevant to one property, neighborhood, or community compared with a different location. This last skillset is particularly important for building and neighborhood design, because their interventions will bring the greatest benefit if they are targeted to the unique combination of needs on and immediately surrounding the project site.

Each element adds an important dimension to understanding how a discipline-specific task such as: designing a health clinic in a low-income urban neighborhood (discipline: architecture); drafting a climate action plan (discipline: planning); or updating a community health needs assessment (discipline: public health) could leverage synergistic action in other disciplines to maximize co-benefits and minimize co-harms to population and planetary health. Students who learn how to use all of these elements in concert with each other will be well positioned after graduation to diagnose and act on hitherto unrecognized leverage points in the climate crisis.

### 2.2. Built environment and public health clearinghouse

This study draws from two openly available clearinghouses, the Built Environment and Public Health Clearinghouse (BEPHC) and the Global Consortium on Climate and Health Education (GCCHE), to scan the landscape of university offerings at the intersection of climate, health, and equity in the built environment (47, 61, 62).

The BEPHC was initially compiled to support the development of the seminal 2009 Botchwey et al. paper described above. Dr. Botchwey confirmed via private correspondence that the original list was manually updated between 2019 and 2021. As part of the update, researchers verified the information in the original list and added universities, programs, courses, and staff names that were gathered through a manual Internet search.

The current website shares information about universities that offered interdisciplinary courses on the links between the built environment and public health at one time, available joint degrees and joint concentrations, as well as model curricula, such as “History and Theory of Architecture + Health (Health and the Built Environment)” taught by Dr. Stephen Verderber at the University of Toronto (63). It also links to relevant openly available datasets.

The degree programs portion of the clearinghouse divides academic offerings into four tiers of content. The higher the tier, the stronger the institutional support for training at the intersection of population health and the built environment (61):

Tier 1: some faculty members have a stated research interest or specialization in the links between human health and the built environment.

Tier 2: at least one course addressing the links between human health and the built environment is offered and may be cross-listed with another department.

Tier 3: at least one interdisciplinary concentration, specialization, certificate, or specialize degree is offered.

Tier 4: joint degree in Master of Public Health and Master of Community or Urban Planning is offered.

The clearinghouse mostly points users to university architecture and planning departments. It occasionally offers links to schools of public health if that is where the joint degree program is housed. But, the website is primarily designed to support students, professors, and university leaders in the design fields.

Information from the 2018 version of the clearinghouse was collected in mid- to late 2018. Information from the revised clearinghouse was collected in October 2022.

### 2.3. Global consortium on climate and health education list of member institutions

The Global Consortium on Climate and Health Education (GCCHE) was founded in 2017 in response to a 2015 pledge that was spearheaded by the US White House and the Columbia University Mailman School of Public Health’s Climate and Health Program and signed by 115 health professions schools from around the world in the lead up to the COP-21 meeting in Paris (64). Its mission is “[t]o unite health professional training institutions, health societies, and regional health organizations to create a global climate-ready health sector, prepared to mobilize and lead health promotion and response in the era of climate change, while restoring the health of the planet.” To that end, the GCCHE recruits health professions schools to publicly endorse climate and health educational offerings in their school by joining the consortium.

Membership in the consortium is mostly limited to schools of medicine, nursing, and public health. The list included in this study focused on schools of public health, because those are the schools that have been more likely in the past to establish joint degree programs with urban and community planning schools.

Information from the 2018 version of the clearinghouse was collected in mid- to late 2018. The 2022 dataset includes the list of member institutions active in October 2022.

### 2.4. Mixed-methods study design

**Figure 2:**
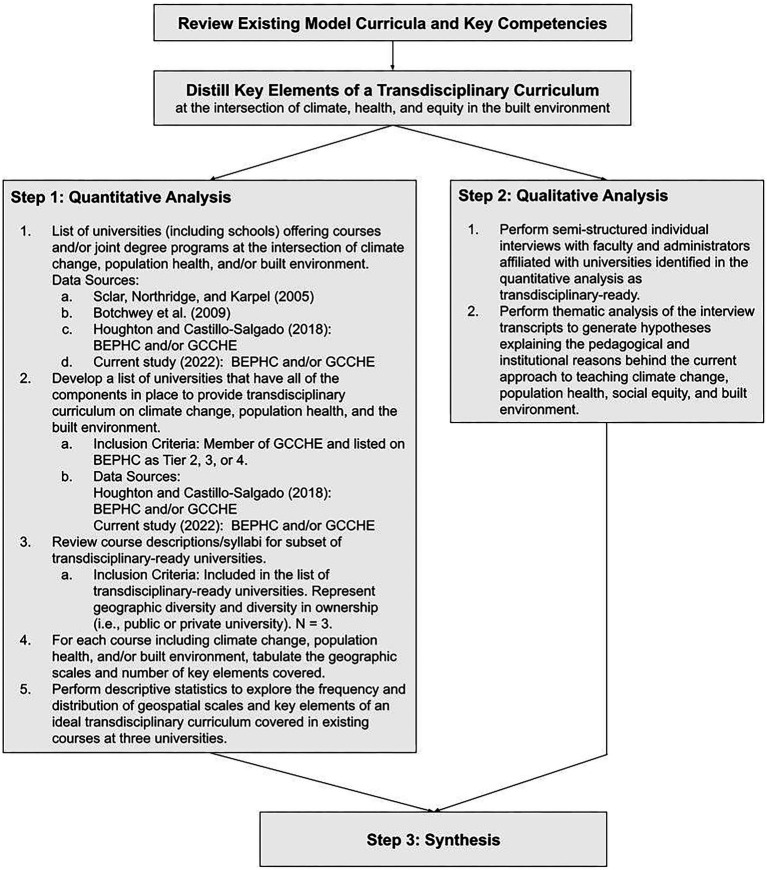
Three-step mixed methods process.

This study used a three-step, mixed-methods process to explore the extent to which all three topics (climate change, population health, and built environment) are integrated into course curricula and the pedagogical and institutional reasons underpinning the current system (Figure 2).

Mixed methods research draws on both quantitative and qualitative methods, combining an assessment of the magnitude or frequency of a phenomenon with an exploration of the meaning behind the quantitative results (65). According to the US National Institutes of Health (65), mixed methods are well adapted to multi-level research questions such as the one posed by this study, which crosses both geospatial levels (e.g., building scale up to global scale) and disciplinary boundaries.

Following a similar method to Sclar, et al. (49) and Botchwey et al. (47), information was collected about degree programs, courses, and commitments made by universities to teach a combination of climate change, population health, and/or the built environment. I followed the quantitative analysis with a set of qualitative, semi-structured interviews with university representatives to generate hypotheses explaining the pedagogical and institutional reasons behind the current system. Finally, I combined the quantitative and qualitative results into a set of synthesized hypotheses about the current extent of training available to students who will graduate into fields where they will be expected to consider the wider systemic implications of their work beyond their home discipline (Figure 2).

### 2.5. Quantitative analysis

**Figure 3:**
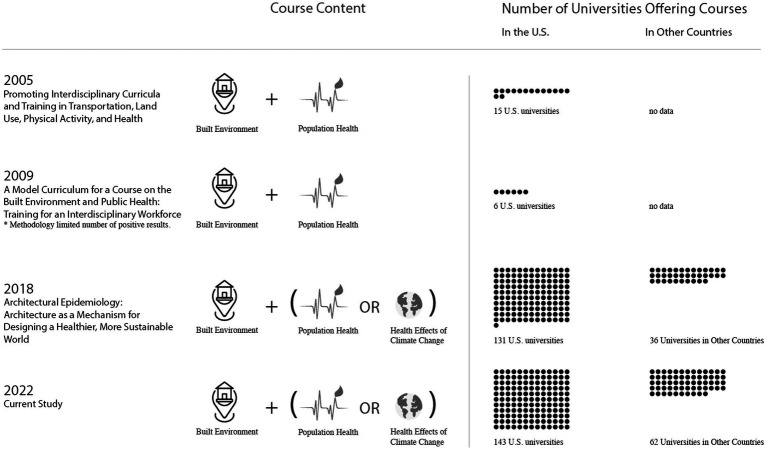
Change in the number of universities teaching courses addressing the relationship between the built environment and population health and/or climate change (2005–2022).

I compiled a list of universities (including schools) offering courses and/or joint degree programs at the intersection of climate change, population health, and/or built environment at four points in time (Figure 3): 2005 (49), 2009 (47), 2018 (66), and 2022 (current study).

The 2018 and 2022 lists drew on the same two curriculum clearinghouses: the Built Environment and Public Health Clearinghouse (BEPHC) (61) and the list of Global Consortium on Climate and Health Education (GCCHE) (64) member institutions. Universities that were both members of GCCHE and listed as Tier 2, 3, or 4 in BEPHC were identified as having all the components in place to provide transdisciplinary curriculum addressing climate, health, and equity in the built environment. These universities are labeled “transdisciplinary-ready” through the remainder of the paper.

In 2022, I reviewed the entire course catalog for the schools of architecture, planning, and public health in three of the 15 transdisciplinary-ready universities: Columbia University, University of Colorado, and University of California at Los Angeles. I selected these universities for review based on the diversity of their geographic locations (East Coast, Mountain West, and West Coast, respectively) and ownership (one private institution and two public institutions, respectively).

For each course including climate change, population health, and/or built environment, I tabulated the geographic scales and number of key elements that were covered. In total, I reviewed 99 course titles and descriptions across the three universities. I also reviewed the course syllabus, if publicly available.

Finally, a research assistant and I performed descriptive statistics to explore the frequency and distribution of geospatial scales and key elements across disciplines. We used Microsoft Excel Version 2023 (Microsoft 365, Redmond, WA) and Stata BE Version 17.0 (StataCorp, College Station, TX) to develop bar charts, frequency tabulations, and chi-square tests.

### 2.6. Qualitative analysis

I used purposeful and snowball methods to recruit professors and administrators from “transdisciplinary-ready” universities to participate in semi-structured interviews in late May and early June 2023. All interviews were conducted over Zoom (San Jose, CA) and analyzed using the video recording and written transcript. The interview guide and consent script adhered to the Harvard T.H. Chan School of Public Health Institutional Review Board (IRB) protocols for non-human subject research. As a result, I only asked participants questions related to the facts about the program at their organization and its development, excluding any questions about personal thoughts and interpretations.

The interview guide was structured to support inductive thematic analysis based on grounded theory (67) – an approach that acknowledges the interviewer’s active participation in the creation of knowledge. Given my active role in each interview, I included safeguards against confirmation bias (i.e., the tendency to focus on evidence supporting one’s existing hypothesis and discount contrary evidence (60)), such as reminding participants not to share their personal opinions and consciously crafting neutral (i.e., non-leading) questions.

### 2.7. Synthesis

I used the results of the thematic analysis to interrogate and frame the results from the quantitative analysis. Of particular interest was the question of whether the clearinghouses’ focus on formal institutional policies and programs might have hidden informal transdisciplinary activity that would benefit from formal institutional support.

## 3. Results

### 3.1. Change in number of multidisciplinary and/or interdisciplinary courses linking climate, health, and equity in the built environment

The number of universities offering multidisciplinary (i.e., multiple disciplines involved, but discipline-specific methods) and/or interdisciplinary (i.e., methods cross disciplinary boundaries to create shared knowledge) curricula linking the built environment with population health has increased substantially since 2005–2009, as shown in Figure 3. Houghton and Castillo-Salgado (66) built on the 2005 and 2009 studies by adding a screen for the health effects of climate change in addition to considering the links between population health and the built environment. From 2018 to 2022, the number of universities in both clearinghouses grew modestly both in the US (increasing from 131 to 143) and in other countries (increasing from 36 to 62).

The reader is advised to consider three important caveats when considering the information in Figure 3. First, neither the BEPHC nor the GCCHE clearinghouse should be considered a comprehensive list. Instead, the righthand column should be read more as an indication that the number of universities offering courses at the intersection of the built environment and either population health or the health effects of climate change appears to be growing both in the US and worldwide. Furthermore, given the fact that only 13 non-US universities appear in the 2022 BEPHC clearinghouse, that set of results should be viewed with particular caution.

Second, many universities included in the BEPHC and GCCHE lists offer more than one relevant course. The total number of courses could therefore be expected to be larger than the number of dots in Figure 3 (which represent universities, not courses).

Third, most of the courses included in the review did not include all three topics (built environment, population health, and climate change). Instead, they addressed the link between the built environment and either population health or the health effects of climate change. This is a major gap that should be central to the conversation about how to develop a truly transdisciplinary course of study addressing climate, health, and equity in the built environment.

### 3.2. Overlap in universities included in both clearinghouses (2022)

**Figure 4:**
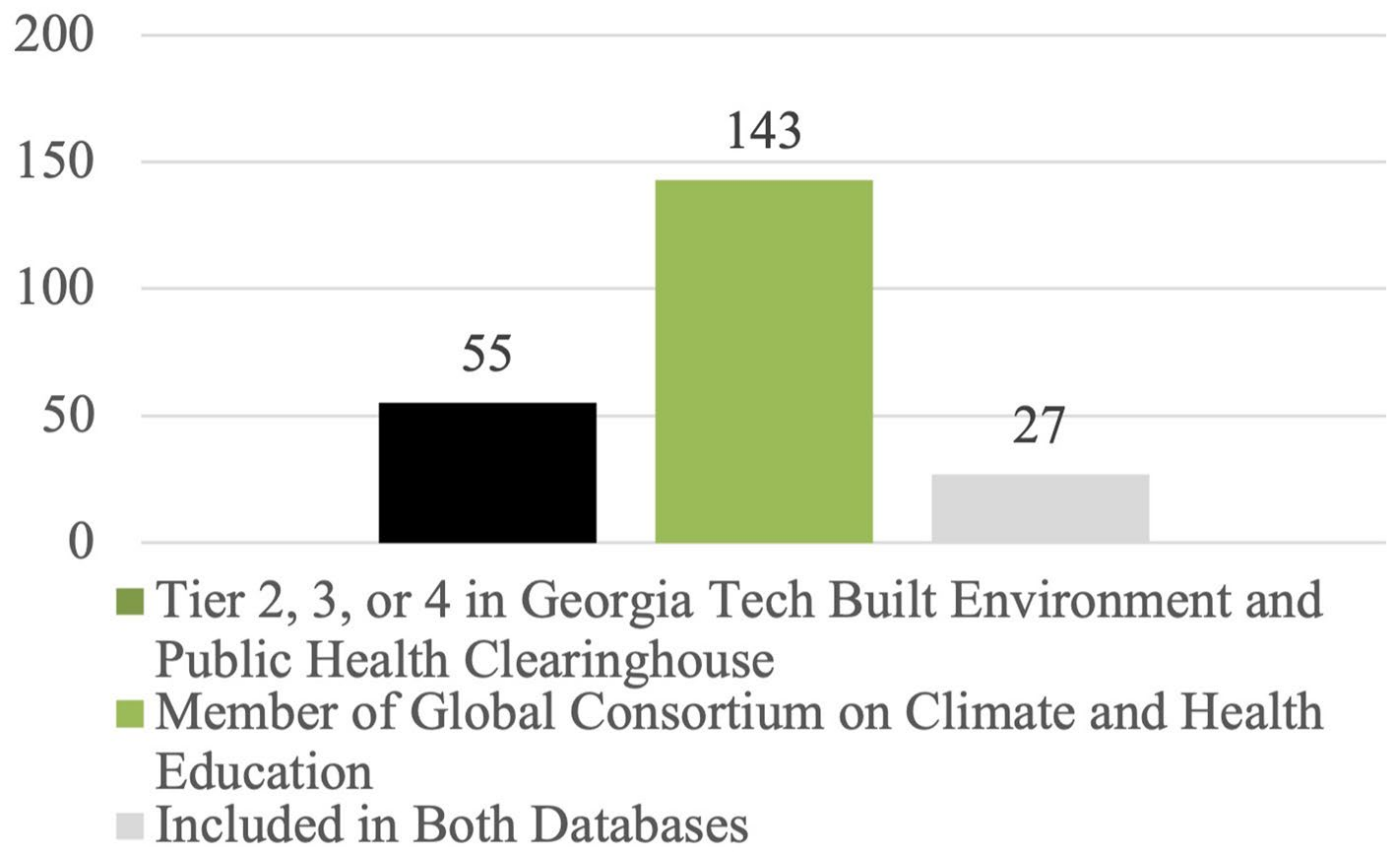
Number of universities listed at Tier 2, 3, or 4 in the built environment and public health clearinghouse, listed as a member of the global consortium on climate and health education, and listed on both databases (*n* = 225 total universities).

Figure 4 illustrates the limited overlap between universities in the BEPHC clearinghouse compared with the GCCHE clearinghouse, in that only 27 of the 55 universities listed at Tier 2, 3, or 4 in the BEPHC clearinghouse (roughly 50%) were also listed as members of the GCCHE.

A note to the reader: it is important to consider that the databases pick up different schools (architecture and planning schools in the BEPHC clearinghouse and schools of public health and medicine in the GCCHE clearinghouse). So, the fact that the same university is included on both lists does not necessarily mean that the two schools or departments realize that they are both claiming leadership on related topics. Instead, they could be considered “transdisciplinary-ready,” having all the key components in place should they wish to provide courses and joint degrees that draw on multiple disciplines and offer practice-based learning opportunities.

### 3.3. Overlap in course offerings linking built environment with population health and/or climate change: sample of course descriptions

**Figure 5:**
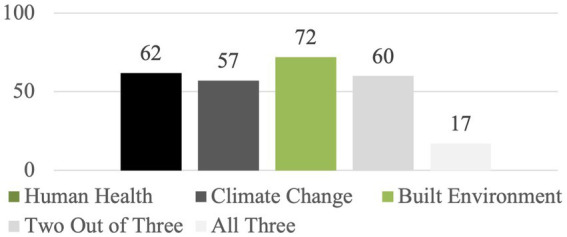
Number of courses in the 3 university sample that address one or more of the following topics: human health, climate change, built environment (*n* = 99 courses across the three universities).

Figure 5 indicates that, of the 99 relevant course titles and descriptions included in the Columbia University, University of Colorado, and University of California at Los Angeles course catalogs, 17 courses addressed all three topics: population health, climate change, and/or the built environment. And, 60 courses addressed two out of the three topics (Figure 5).

**Figure 6:**
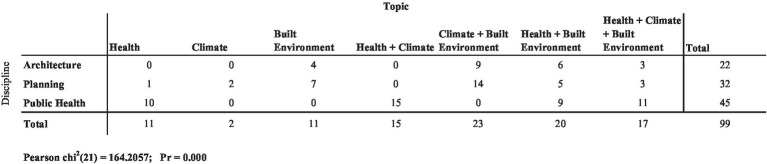
Frequency and distribution of course topics by discipline (*n* = 99 courses across the three universities).

When organized by discipline, we see that the majority of courses covering all three topics (*n* = 11) were located in schools of public health, with architecture and planning schools each offering three courses covering all three topics. The chi-square test for the frequency estimate was statistically significant, *X*^2^(21, *n* = 99)164.206, *p* < 0.0001 (Figure 6).

**Figure 7:**
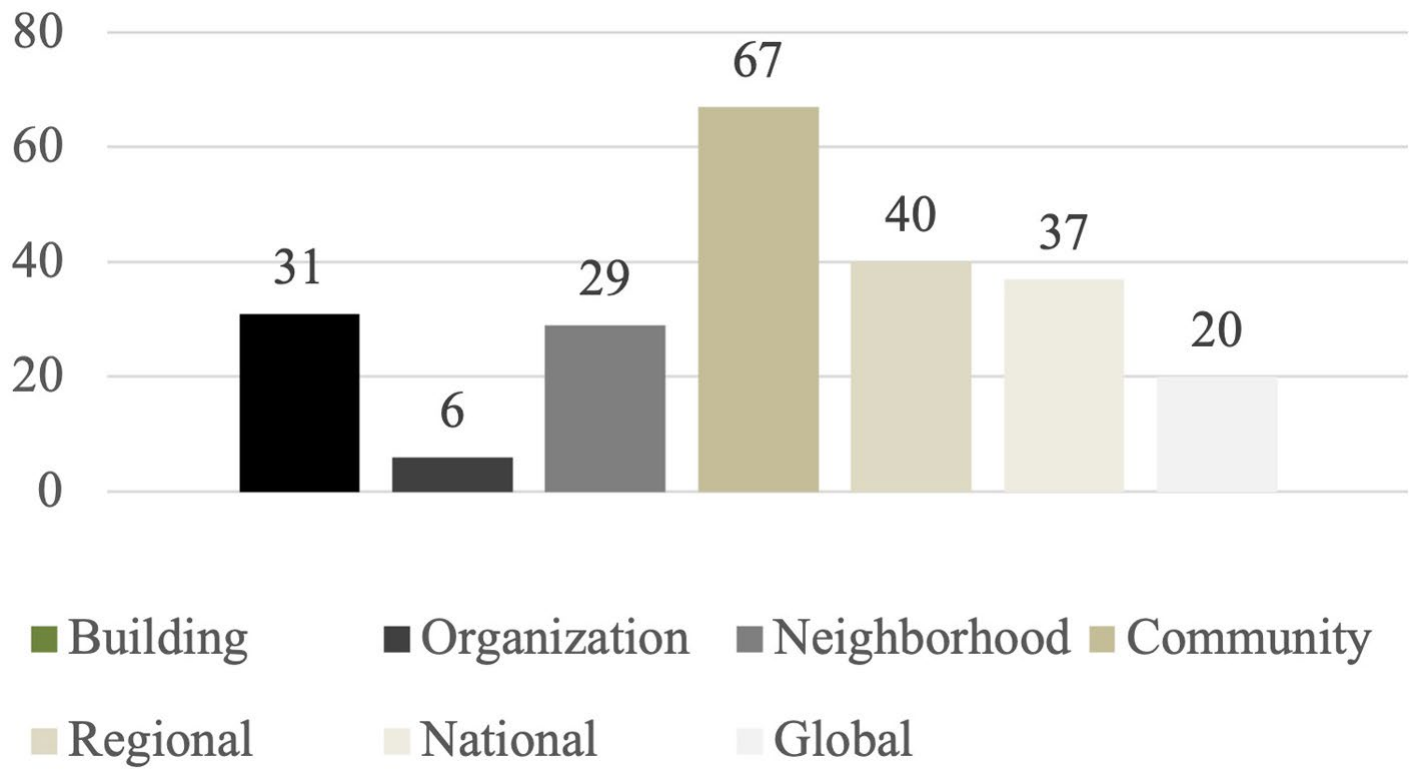
Geographic scale of courses (*n* = 99 courses across the three universities).

While the majority of courses in the three-university sample (68) covered the links between the built environment and population health/climate change at the community scale, most courses touched on multiple scales – including 37% (*n* = 37) considering the building or organization scale at least to some extent (Figure 7).

**Figure 8:**
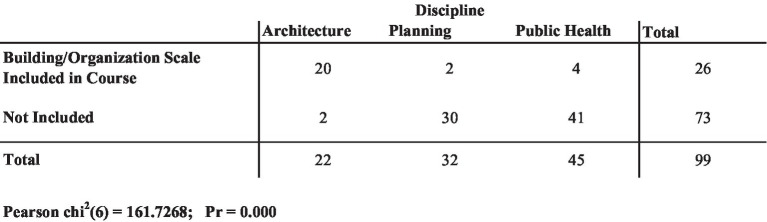
Geographic scale of courses (*n* = 99 courses across the three universities).

Unsurprisingly, courses in architecture schools were much more likely than planning or public health schools to cover the building or organization scale. Figure 8 shows that 20 out of 22 courses in architecture schools met that criteria, compared with only two out of 32 courses in planning schools and four out of 45 courses in public health schools. The chi-square test for the frequency estimate was statistically significant, *X*^2^(6, *n* = 99)161.727, *p* < 0.0001 (Figure 8). Given the fact that there were so many fewer architecture courses in the dataset compared with planning and public health, the fact that the building and organization scale are so often excluded from the curriculum in planning and public health schools further exacerbates the gap in opportunities for students to learn about the pivotal role that buildings play in a transdisciplinary approach to climate, health, and equity in the built environment.

**Figure 9:**
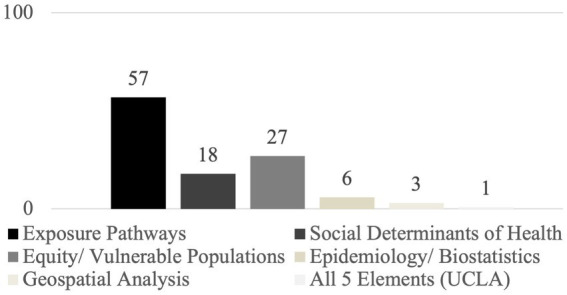
Key course elements across the sample dataset of course titles and descriptions (*n* = 99 courses across the three universities).

Figure 9 compares the same sample set of 99 course descriptions to the five key elements described in the Introduction. The majority of courses (*n* = 57) explicitly address the built environment as an exposure pathway for health outcomes – whether related to chronic disease, climate change, or another public health topic. Almost one third (*n* = 28) center equity and/or vulnerable populations in the topics covered by the course. But, the remaining key course components (social determinants of health, epidemiology/biostatistics methods, and geospatial analysis) were not comprehensively addressed by most courses. Only one course, EHS C200B Foundations of Environmental Health Sciences for Public Health Professionals at UCLA, included all of the key course components for a transdisciplinary curriculum linking the built environment to population health and the health effects of climate change.

**Figure 10:**
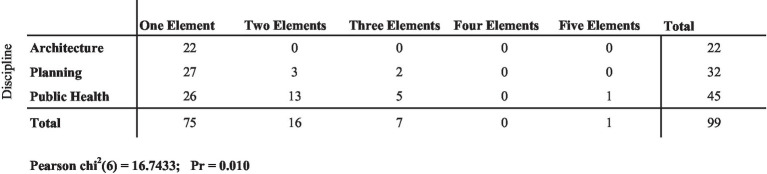
Number of key elements per course, by discipline (*n* = 99 courses across the three universities).

All of the architecture courses in the sample set of 99 course descriptions included only one key course element: either exposure pathways or equity/vulnerable populations. Planning schools followed close behind architecture schools, offering only three courses with two elements (exposure pathways and equity/vulnerable populations) and two courses with three key elements (exposure pathways, equity/vulnerable populations, and social determinants of health). Public health schools were by far the most likely to include two elements (mostly exposure pathways and equity/vulnerable populations) (*n* = 13). Five public health courses included three elements. And, the course at UCLA covering all five elements (mentioned above) was housed in the school of public health. The chi-square test for the frequency estimate was statistically significant, *X*^2^(6, *n* = 99)16.743, *p* = 0.010 (Figure 10).

### 3.4. Joint degree programs

The BEPHC clearinghouse identified 15 universities with joint degree programs in urban planning and public health in 2022 (Table 2). Four of the universities on the list were also identified as offering joint degrees in the Sclar and Northridge report from 2005: Columbia University, University of California Berkeley, University of Michigan, and University of North Carolina at Chapel Hill. Five joint degree programs (1/3 of the total) include the health effects of climate change as part of the required course of study. In all cases, this topic is covered in a course or courses taught in the school of public health.

**Table 2:** Joint master degrees in urban planning and public health: 2022 results.

University	Required Course Addressing Climate Change and Health?
Columbia University*	No
Florida State University	No
Harvard University	No
Portland State University	No
Queens University	No
University of California, Berkeley*	PH 271G Global Climate Change and Public Health
University of California, Los Angeles (UCLA)	EHS C200B Foundations of Environmental Health Sciences for Public Health Professionals
	EHS 208 Built Environment and Health
University of Colorado, Denver	No
University of Illinois at Urbana-Champaign	No
University of Maryland	No
University of Michigan*	No
University of Minnesota	PubH 6,250 Foundations of Public Health
University of North Carolina at Chapel Hill (UNC)*	ENVR 600 Environmental Health
University of Southern California	No
University of Washington	PHI 511 Foundations of Public Health
	ENVH 501 Foundations of Environmental and Occupational Health
	URBDP 538/ENVH 538 Public Health and the Built Environment

*Indicates this degree was also offered in 2005.

Since the BEPHC clearinghouse is primarily focused on community and urban planning programs, it did not specify whether any joint degrees in public health were offered in collaboration with architecture programs. To fill this gap, I performed a Google search in September 2022 using the terms “master of public health,” “public health,” and “architecture” – returning no joint Master of Architecture/Master of Public Health (MArch/MPH) degree programs. I followed up the general search query by visiting the websites of 15 architecture schools included on the National Architectural Accrediting Board list of accredited schools (68). None of the schools offered a joint degree or concentration in architecture and public health.

From September to November 2022, I asked ten academicians in US universities whether their university had established or was considering establishing a joint MArch/MPH degree in collaboration with a school of public health or health science center. A professor at a public university responded that their school of architecture and health science center were in the early stages of conversations about establishing a joint degree. But, the remaining responses pointed either to a joint degree or concentration between the school of public health and the department of urban or community planning, an MArch degree with a concentration in the design of healthcare facilities (such as the Architecture + Health concentration at Clemson University (69)), or individual courses touching on the links between building design and population health outcomes or climate change.

In order to launch a comprehensive, transdisciplinary program on climate, health, and equity in the built environment, universities will need to offer courses, concentrations, and joint degree programs that are open to students from multiple departments (at a minimum: architecture, planning, and public health); explore the links between the built environment, population health, social equity, and climate change; and, incorporate project-based learning as part of the curriculum.

Table 3 lists the universities in the BEPHC and GCCHE clearinghouses that met all or all but one of these structural components. 18 universities met all of the components in both 2018 and 2022. 14 universities have all but one of the components in place to launch a truly transdisciplinary curriculum. In both sets of universities, an institution’s level of transdisciplinary-readiness is tempered by its designated tier in the BEPHC clearinghouse. To clarify the stratification across institutions, the righthand column in Table 3 lists additional institutional supports that would strengthen a university’s position as a transdisciplinary-ready institution: such as moving from Tier 3 in the BEPHC clearinghouse (interdisciplinary concentration, specialization, certificate, or specialized degree) to Tier 4 (joint degree program).

**Table 3:** Universities with all or almost all of the components in place to launch a comprehensive, transdisciplinary program on climate, health, and equity in the built environment (2018–2022).

Institutions listed in BEPHC and GCCHE clearinghouses in both 2018 and 2022	Relevant School/Department(s)	BEPHC Tier*	Adding the following elements would move the institution closer to a transdisciplinary curriculum
Columbia University	Graduate School of Architecture Planning and Preservation	Tier 4	
	Mailman School of Public Health		
Harvard University	Graduate School of Design	Tier 4	
State University of New York at Buffalo	School of Architecture and Planning	Tier 2	Offer a concentration or joint degree.
	School of Public Health and Health Professions		
Temple University	School of Environmental Design	Tier 3	Offer a joint degree.
	College of Public Health		
Texas A&M University	College of Architecture	Tier 3	Offer a joint degree.
	School of Public Health		
University of California at Berkeley (Berkeley)	College of Environmental Design	Tier 4	
	School of Public Health		
University of California at Los Angeles (UCLA)	UCLA Fielding School of Public Health	Tier 4	
	Luskin School of Public Affairs		
University of Colorado	College of Architecture and Planning	Tier 4	
	Colorado School of Public Health		
University of Illinois at Chicago	School of Public Health	Tier 2	Offer a concentration or joint degree.
	Department of Urban and Regional Planning		
University of Illinois at Urbana-Champaign	College of Urban Planning and Public Affairs	Tier 4	
	Master of Public Health Program		
University of Michigan	Taubman College Architecture + Urban Planning	Tier 4	
	School of Public Health		
University of Minnesota	Humphrey School of Public Affairs	Tier 4	
	School of Public Health		
University of North Carolina at Chapel Hill (UNC)	Department of City and Regional Planning	Tier 4	
	Gillings School of Global Public Health		
University of Oklahoma	College of Architecture	Tier 2	Offer a concentration or joint degree.
	Health Sciences Center – College of Public Health		
University of Southern California	Sol Price School of Public Policy	Tier 4	
	Master of Public Health Program		
University of Toronto**	Geography and Program in Planning	Tier 3	Offer a joint degree.
	Dalla Lana School of Public Health		
University of Washington	Urban Design and Planning; Environmental & Occupational Health Sciences; Health Services	Tier 4	
	School of Public Health		
University of Waterloo**	School of Planning	Tier 2	Offer a concentration or joint degree.
	School of Public Health and Health Systems		
Institutions Meeting All But One Structural Criteria in 2022			
Boston University	Boston University Metropolitan College	Tier 2	Partner with the architecture department at a peer institution to offer courses linking architectural design to climate change, population health, and equity. Offer a concentration or joint degree with public health.
	School of Public Health		
Florida State University	Department of Urban and Regional Planning	Tier 4	Offer courses linking architectural design to climate change, population health, and equity. Join the Global Consortium on Climate and Health Education.
	College of Social Sciences and Public Policy, School of Public Health		
George Washington University	College of Professional Studies	Tier 2	Offer courses linking architectural design to climate change, population health, and equity. Offer a concentration or joint degree with public health.
	Milken Institute School of Public Health		
Hunter College of the City University of New York	Hunter College Urban Affairs and Planning	Tier 2	Add the health effects of climate change to the curriculum linking architecture and planning to population health. Offer a concentration or joint degree with public health. Join the Global Consortium on Climate and Health Education.
Ohio State University	Knowlton School of Architecture		Removed from the BEPHC clearinghouse website between 2018 and 2022.
	School of Health and Rehabilitation Sciences		
	College of Medicine		
Ryerson University/Toronto Metropolitan University**	School of Urban and Regional Planning		Removed from the BEPHC clearinghouse website between 2018 and 2022.
	School of Occupational and Public Health		
State University of New York at Albany	College of Arts and Sciences: Geography and Planning	Tier 3	Offer courses linking architectural design to climate change, population health, and equity. Offer a joint degree.
	School of Public Health		
University of Arizona	College of Architecture + Planning + Landscape Architecture		Removed from the BEPHC clearinghouse website between 2018 and 2022.
	Mel and Enid Zuckerman College of Public Health		
University of Iowa	School of Urban and Regional Planning	Tier 2	Offer courses linking architectural design to climate change, population health, and equity. Offer a concentration or joint degree with public health.
	College of Public Health		
University of Massachusetts Amherst	Landscape Architecture and Regional Planning		Removed from the BEPHC clearinghouse website between 2018 and 2022.
	School of Public Health and Health Sciences		
University of Memphis	College of Arts and Sciences		Removed from the BEPHC clearinghouse website between 2018 and 2022.
	School of Public Health		
University of Nebraska	UN Medical Center College of Public Health – Omaha		Removed from the BEPHC clearinghouse website between 2018 and 2022.
	College of Architecture Creating Spaces – Lincoln		
University of New Mexico	School of Architecture and Planning		Removed from the BEPHC clearinghouse website between 2018 and 2022.
	Health Sciences Center		
University of Pennsylvania	School of Design		Removed from the BEPHC clearinghouse website between 2018 and 2022.
	Master of Public Health Program		

Components: courses offered in architecture, planning, and public health schools; coursework linking climate change, population health, and social equity in the built environment and supported through a joint degree (Tier 4), concentration (Tier 3), or individual courses (Tier 2). The higher the Tier, the more structural components are in place at the intersection of population health and the built environment. *Curriculum Tier according to Georgia tech built environment and public health clearinghouse. **Located in Canada (All other universities on the list are located in United States).

### 3.5. Qualitative analysis of pedagogy and institutional approaches to transdisciplinary courses and joint degrees on climate, health, equity and the built environment

I conducted ten key informant interviews with faculty and administrators at seven transdisciplinary-ready universities after completing the quantitative phase of the study. I used the opportunity to ask participants, many of whom are pioneers in the creation of university courses and joint degree programs at the intersection of the built environment and health, why these topics remain at the margins of all three disciplines: architecture, planning, and public health. The seven institutions in the dataset span four geographic regions in the US, vary in size, and include both public and private institutions. Using inductive thematic analysis, I elicited four major themes explaining current pedagogy and administrative priorities in US-based transdisciplinary-ready universities.

### 3.6. Accreditation

All of the interview participants stated that accreditation bodies play a pivotal role in moving areas of study from the margins to the center of academic curricula. A major barrier to institutionalizing transdisciplinary curricula addressing climate, health, and equity in the built environment is that one or more of those topics are not identified as core competencies in the named disciplines in the study (architecture, planning, and public health). For example, climate change is not listed as a core competency by the Public Health Accreditation Board (70) and the health effects of climate change are not listed as core competencies by either the National Architectural Accreditation Board (38) or the Planning Accreditation Board (71). Three participants described using their own research and/or status in their home institution to champion inclusion of climate change, population health, and/or social equity in the built environment in core courses – such as survey courses and core design studios. For example, one participant shared that they were able to integrate population health into a core course in a school of architecture as a result of “a series of alignments between an interim director, and … a few senior faculty [on the curriculum committee] who said, ‘You’ve convinced us.’” The participant added, “It took me [a few] years of standing up in meetings and … [saying], ‘We’re doing something that other schools aren’t doing. Please support it.’”

All participants pointed to the need to institutionalize the approach in order to ensure its longevity. Currently, even in universities with longstanding programs, interested faculty and administrators expressed a sense of fragility. They questioned whether even joint degree programs will survive after the current crop of faculty retires. One participant shared, “You know, I’m going to put health at the top of the list for an architecture course. But it’s going to compete with every other topic that every other faculty member thinks is the most important thing anybody ought to be doing. … And so long as it’s optional, you’ll get a scattering of courses around the country. They are generally going to be based on the interest of the faculty willing to teach them. When that faculty retires, [the university is] not particular about a hire to replace [them], because [the topic] was never made central.”

Another participant expressed frustration with the university and accreditation structures that stand in the way of change: “There’s a lot of inertia. And I feel like I’ve been the lone or nearly the lone person trying to make these things happen for 10 years. And when a new administrator comes in and they do not seem to be at all interested in supporting [transdisciplinary or cross-listed courses]. … That’s when I just get frustrated. And I’m like, okay. I’m this many years from retirement. I do not have a lot more to expend here.” A third participant stated that a course remains on their university’s website in spite of the fact that it has not been taught since the professor who created and delivered it retired.

### 3.7. Budgets and finances

Most participants stated that cross-listing courses – particularly across different schools at a university, but sometimes even across different departments within the same school – can be a challenge because school (and sometimes departmental) budgets are partially determined by student enrollment. In other words, if 10 students enroll in a cross-listed course using the school of architecture and planning code but only five students enroll using the school of public health code, the school of architecture and planning will receive two thirds of the enrollment funding even if classes are hosted in a classroom at the school of public health. This kind of incentive structure (coupled with bureaucratic hurdles, such as different grading systems in different schools) can create a real barrier to professors’ collaborating to develop and deliver transdisciplinary courses or joint degree programs. It is easier to create a joint degree program that passes the student back and forth – so that she pays for courses in the planning department one year and courses in the school of public health the next year, for example. One participant shared a story about a graduate student who wanted to complete both a Master of Urban Planning (MUP) and a Master of Public Health (MPH). According to the interviewee: “It took three and a half years, because it was about money and revenue. She had to do the MUP and MPH separately. … It was because they wanted her to pay separately [for the two degrees].” Needless to say, those kinds of practical decisions do not necessarily optimize the student’s learning opportunities.

Every participant agreed that an effective way to overcome the financial and bureaucratic barriers to transdisciplinary courses is for the university to establish a superstructure of sorts that offers funding, streamlined course approvals, and other support systems that lift transdisciplinary conversations out of the departmental level and up to a university level, where topics like climate change can be promoted by high ranking administration officials, such as the Provost or Chancellor. In 2022, Harvard University announced the creation of the Salata Institute for Climate and Sustainability, which is overseen by the Vice Provost for Climate and Sustainability. The Institute is designed as a university-wide initiative aimed at supporting “comprehensive University-wide education in climate and environmental fields” (72). Several interview participants expressed optimism that this experiment in university-wide efforts to tackle climate change might offer a possible solution to the budget and financing barriers to performing transdisciplinary work at Harvard.

Other universities have attempted to circumvent school-specific budgetary siloes by creating small research grant programs and administrative support for developing transdisciplinary curricula. Unfortunately, these efforts often hit glass ceilings unless the university creates an infrastructure supporting their implementation. As one participant explained, “The college got a grant from the Chancellor’s office to create [a cross-listed course]. … I spent [several] years doing a market study, working with the person who was in charge of the program in public health at that time. She was very committed to the collaborative effort. But, ultimately, she wasn’t permanent. She was a contingent faculty member who had been put in charge because somebody retired or something. She also was the one who said, ‘There’s just no room in what we are doing [because of accreditation requirements].’”

Another participant described multidisciplinary grants issued by the university and requiring two or more schools to participate as an informal workaround that only involves students as research assistants. That interviewee observed that research assistants often do not make the connection about the links across disciplines by “coincidence” or “exposure.” They continued: “I feel like education is a lot like the healthcare system. The burden of synthesis and coordination is on the student. … The curriculum is not working to help [the student] figure it out. … We started maybe a year back trying to develop a concentration [linking population health and the built environment], just identifying all of the courses across the university. … The burden of even putting together [an inventory of courses] was on the student. … [It’s such a big lift that] there’s an incentive to only showcase courses [in the concentration] that are in or adjacent to [the home discipline].”

### 3.8. Informal workarounds

Given the institutional and accreditation barriers to centering climate, health, and equity in the built environment in any given discipline, all of the interview participants described informal workarounds they use to introduce these topics into their research and interaction with students.

In many institutions, professors seek out like minded colleagues in different departments to jointly apply for research funding and deliver guest lectures in each other’s courses. As one professor put it, “I cultivate relationships with faculty members [in other schools], partly because we work on the sustainable campus effort that brings the physical world front and center through the campus and structures. I serve on advisory committees and … doctoral committees. And, [professors from other schools and I] lecture in each other’s courses. We always know that we are going to make something happen at the personal level [even if there is no formal partnership between the two schools].”

The goal of these informal partnerships is to expose students to courses and professors in other disciplines, so that it is easier for students interested in these topics to find like-minded professors. Participants showed less consensus around whether students were likely to connect the dots on their own without support from faculty members. One participant stated, “I actually feel pretty strongly that students can do that work to synthesize [the intersection between health and the built environment] on their own. And, we do not have to design it for them every time.” Another participant gave an example of the kinds of linkages students are expected to make without professorial support: “The students are getting more about climate change through, for example, their MEP courses and some of the other courses, and so it in some ways it is left up to them to make the bridge [between climate change and health].” A third participant shared, “I have observed that many students [particularly undergraduates] do not connect the dots on their own. It requires a professor to show them that the work they are doing links over to work happening in another department.”

Professors use case studies and student projects to create opportunities for students to integrate disparate concepts learned earlier in the course into a synthesized response to a complex challenge. Practice-based learning projects add the component of learning from community stakeholders – a key component to centering social equity in transdisciplinary work. These courses and research opportunities face similar challenges to the elective courses described above. Unless they are institutionalized as part of the core curriculum, they are experienced as one-off projects, requiring additional work and returning questionable rewards to the professor or administrator who went to the effort to set them up. One participant shared, “The informal process [of connecting students with professors in different schools] serves my students well for the most part, because I know where to send them. … But [each connection] is a one-off, and it’s not a very effective. … It’s haphazard.”

### 3.9. Role of the student, role of the university

Returning to Scholz’s critique of the university (10), an area of disagreement among participants involved the role of the university in setting an agenda for the future of transdisciplinary research and pedagogy. Some participants reflected their institution’s observation that students had been asking for transdisciplinary training for years – particularly around environmental sustainability, social equity, and health. One participant shared the observation: “It’s not that there’s not enough student interest. It’s really that the professors aren’t interested …. The students do amazing things. And most of them do pick up on [the links between design, population health, and social equity]. There’s a core group of [undergraduates] that actually come back to our graduate program because they are really interested in it, and they know that we have this specialty.”

Other institutions did not see the same level of interest from prospective students. One participant observed, “You’re never going to have that many students who want to do a full transdisciplinary degree. We actually have a hard enough time getting enough students to fill a class. … But there is a demand for a mix and match way of having these two degrees [MUP/MPH].”

Still others described climate change as an existential threat. But, rather than emphasize climate change in the undergraduate core curriculum, the institution decided to introduce a transdisciplinary climate change and health program at the doctoral level. As a professor from that institution put it, “We do not see that many researchers being turned out who have the skill set that’s going to be needed to address the most ‘wicked’ problems [like climate change]. But, we need people who have the skills to address them. We recognize that it’s a gap in our curriculum. And, we have students who are coming out of the woodwork saying, ‘We want to be part of this solution by doing research in this area.’” A participant from a different institution observed: “Part of [the purpose behind establishing transdisciplinary research and pedagogy] more formally is a signaling process to society. [We are using the university as a platform to communicate that] these are critical issues that need to be rethought.”

In sum, given the difficulties in institutionalizing any kind of transdisciplinary research or pedagogy, the fact that the number of courses and joint MUP/MPH degrees addressing climate, health, and equity in the built environment appears to be growing may reflect a larger shift in societal priorities that will lead to institutional reforms at universities over time.

## 4. Discussion

### 4.1. Historical precedents, future needs

A review of the BEPHC and GCCHE clearinghouses, web search, and semi-structured interviews with US professors and administrators revealed a strong history of champions within universities who have established joint degrees and worked informally with colleagues in different departments and schools to advance the state of knowledge and provide students with training on the intersection of climate change, population health, and social equity in the built environment.

This study revealed that four universities have offered joint degrees in planning and public health since at least 2005 (Table 2): Columbia University, University of California Berkeley, University of Michigan, and UNC. Furthermore, five of the universities currently offering joint degrees include the health effects of climate change in at least one required course in the MPH curriculum (Table 2): University of California Berkeley, UCLA, University of Minnesota, UNC, and University of Washington. This is an encouraging sign of the longstanding influence that pioneering researchers on the links between the built environment and population health have had on the fields of public health and community and urban planning. Notably, the book *Making Healthy Places* (62) edited by Drs. Andrew Dannenberg, Howard Frumkin, and Richard Jackson and released in 2011 has served as a textbook for courses following the proposed curriculum in the Botchwey et al. paper from 2009, which included Dr. Dannenberg and Dr. Jackson as co-authors. Furthermore, all three editors are or have been affiliated with two of the universities that both offer dual degrees and are well-positioned to launch a transdisciplinary program on climate, health, and equity in the built environment: UCLA and University of Washington. A new edition of the book, headlined by Dr. Botchwey, was released in 2022 in an expression of optimism that demand for this type of training remains strong (37).

Also striking is the geographic diversity and strong representation of public universities in the cohort of 32 institutions that have all or all but one of the structural components in place to launch a truly transdisciplinary program on climate, health, and equity in the built environment. These universities are located in 19 states and the District of Columbia, including conservative-leaning states like Iowa, Nebraska, Oklahoma, and Texas. Three universities are located in Ontario, Canada: Toronto Metropolitan University, University of Toronto, and University of Waterloo. 27 of the 32 institutions in the cohort (84%) are public universities.

While these universities have taken steps to establish the structural components necessary to launch a transdisciplinary program on climate, health, and equity in the built environment, it is far from clear that students, professors, and research faculty engage in these topics in a transdisciplinary manner. More is required than simply removing structural barriers to transdisciplinary education and research. Other actions will be needed, such as overcoming the disciplinary boundaries that are so entrenched in many universities; cross-listing courses in more than one school; establishing joint compensation mechanisms for transdisciplinary professors and researchers; incentivizing the creation of transdisciplinary courses, student projects, and research grant applications; and, celebrating early adopters of this new approach to education and research.

Eighteen years after the Sclar, Northridge, and Karpel report surfaced many of the same barriers to transdisciplinary programs (49), it is far from clear that these actions have been taken at any institution – even the eleven universities offering joint degrees and identified as having all the necessary structural components in place: Columbia, Harvard, Berkeley, UCLA, University of Colorado, University of Illinois at Urbana-Champaign, University of Michigan, University of Minnesota, UNC, University of Southern California, University of Washington.

The qualitative interviews with ten professors and administrators at seven transdisciplinary-ready institutions revealed informal partnerships filling the gaps that have been created by institutional barriers and the absence of a mandate in the form of accreditation boards. Many of the original pioneers who pushed for integration of built environment considerations into public health curricula and the integration of population health considerations into architecture and planning curricula sit on the verge of retirement and worry that the courses and joint degrees they championed may retire alongside them unless they are folded into the core curriculum in their home disciplines.

### 4.2. Architecture: a key missing element

Both the quantitative and qualitative portions of this study concluded that schools of architecture remain largely excluded from transdisciplinary curricula addressing climate, health, and equity in the built environment. This is a troubling finding, because building design is the place where neighborhood and community plans are either implemented or not. By excluding architects, real estate developers, and other building professionals, joint degree programs and concentrations are missing the key piece that will allow future practitioners to bridge this “last mile” problem and ensure that holistic community plans are built out – and therefore able to achieve their goals related to climate mitigation, climate adaptation, population health outcomes, and social equity.

### 4.3. Limitations

This study faced several limitations. First, the BEPHC and GCCHE clearinghouses list university names and, to some extent, the names of schools and departments. However, they do not provide up to date information. And, they do not link directly to course catalogs. Many universities do not make their syllabi public. And, some do not make their course descriptions public for active courses. As a result, the process of linking the presence of a university on one of the clearinghouse lists to course content was laborious and riddled with missing data.

Neither clearinghouse claims their content to be comprehensive. The BEPHC list was compiled manually using an Internet search. In order for a university to appear on the GCCHE list, that institution must opt in to becoming a member of the consortium. As a result, none of the results in this study should be construed as representative of the current state of transdisciplinary curriculum on climate, health, and equity in the built environment. It simply provides an indication of the rapid growth in joint programs, concentrations, and courses at the intersection of these topics. It also presents a group of transdisciplinary-ready institutions with the opportunity of establishing a learning network that could accelerate the transition towards a more effective approach to education and research on complex, global topics like climate change.

## 5. Conclusion

This study brings the most comprehensive clearinghouses on built environment, population health, and climate change curricula together for the first time to assess the extent to which some postsecondary institutions have all of the pieces in place that would be needed to create the truly transdisciplinary curriculum that is needed to train the future leaders who will usher the world into a post-carbon future.

It found that the number and geographic distribution of courses addressing the built environment, population health, and/or climate change have dramatically increased between 2005–2009 and 2022. However, the overwhelming majority of courses present material only at a conceptual level. The review identified only a handful of joint degrees – all of which link the school of public health with a community/urban planning degree. No joint degree programs were identified linking a public health degree with an architectural degree.

The results of this study show that, while universities are starting to respond to pressure to provide transdisciplinary courses and joint degree programs at the intersection of the built environment, climate change, population health, and social equity, these offerings remain too limited in scope and too conceptual in content as yet to produce graduates who fully possess the necessary research methods and technical skills required to design and implement built environment interventions that will address the needs of society in a changing climate.

## Author contributions

AH conceived the idea for the study and conducted all aspects of research, writing, and editing.

## Conflict of interest

AH is President of Biositu, LLC. She declares that the research was conducted in the absence of any commercial or financial relationships that could be construed as a potential conflict of interest.

## Publisher’s note

All claims expressed in this article are solely those of the authors and do not necessarily represent those of their affiliated organizations, or those of the publisher, the editors and the reviewers. Any product that may be evaluated in this article, or claim that may be made by its manufacturer, is not guaranteed or endorsed by the publisher.
